# Differences in Sirtuin Regulation in Response to Calorie Restriction in *Cryptococcus neoformans*

**DOI:** 10.3390/jof4010026

**Published:** 2018-02-18

**Authors:** Tejas Bouklas, Lindsey Masone, Bettina C. Fries

**Affiliations:** 1 Department of Biomedical Sciences, Long Island University-Post, Brookville, NY 11548, USA; lindsey.masone@my.liu.edu; 2 Department of Medicine (Division of Infectious Diseases) and Department of Molecular Genetics and Microbiology, Stony Brook University, Stony Brook, NY 11794 USA; bettina.fries@stonybrookmedicine.edu

**Keywords:** fungus, pathogen, sirtuins, aging, resilience, glucose

## Abstract

*Cryptococcus neoformans* successfully replicates in low glucose in infected patients. In the serotype A strain, H99, growth in this condition prolongs lifespan regulated by *SIR2*, and can be modulated with *SIR2*-specific drugs. Previous studies show that lifespan modulation of a cryptococcal population affects its sensitivity to antifungals, and survival in an infection model. Sirtuins and their role in longevity are conserved among fungi; however, the effect of glucose starvation is not confirmed even in *Saccharomyces cerevisiae*. Lifespan analysis of *C. neoformans* strains in low glucose showed that 37.5% exhibited pro-longevity, and lifespan of a serotype D strain, RC2, was shortened. Transcriptome comparison of H99 and RC2 under calorie restriction demonstrated differences, confirmed by real-time PCR showing that *SIR2*, *TOR1*, *SCH9*, and *PKA1* expression correlated with lifespan response to calorie restriction. As expected, RC2*-sir2Δ* cells exhibited a shortened lifespan, which was reconstituted. However, shortened lifespan from calorie restriction was independent of *SIR2*. In contrast to H99 but consistent with altered *SIR2* regulation, *SIR2*-specific drugs did not affect outcome of RC2 infection. These data suggest that *SIR2* regulation and response to calorie restriction varies in *C. neoformans*, which should be considered when Sirtuins are investigated as potential therapy targets for fungal infections.

## 1. Introduction

*Cryptococcus neoformans* is a fungal pathogen that predominantly affects immunocompromised individuals, such as HIV/AIDS patients, in whom it causes chronic meningoencephalitis (CME). Patients with this diagnosis primarily die from CME, with the disease following either an acute infection or reactivation of a latent infection [1]. *C. neoformans*’ ability to evade host immunity combined with its ability to replicate in the host poses a challenge to effective recovery. Despite the availability of anti-retroviral and anti-fungal therapy to circumvent these challenges, more than 80% mortality is seen in over 220,000 people affected by cryptococcosis annually [2].

*C. neoformans* is a haploid fungus that predominantly reproduces asexually during its course of infection [3]. Specifically, it undergoes asymmetric mitotic divisions, the sum of which determines its replicative lifespan (RLS) [4]. During these divisions, aging mother cells demonstrate phenotypic changes that include an increased cell size, which has been described in *Saccharomyces cerevisiae*, *Candida albicans*, *Schizosaccharomyces pombe* [5,6,7], and documented for *C. neoformans* by our laboratory [8,9]. Similar to these fungi, old *C. neoformans* cells cease division when RLS is completed.

Several factors have been implicated in the regulation of RLS, the most widely described and oldest intervention being calorie restriction (CR). Studies had originally shown that nutrient sensors, termed sirtuins [10], mediate this process; in fact, in *S. cerevisiae*, an extra copy of *SIR2* increases RLS, whereas its loss decreases RLS [11]. However, this has since been challenged [12], primarily because longevity of yeast mother cells can be altered by affecting levels of calorie restriction (CR). Specifically, these studies have concluded that, in *S. cerevisiae*, Sir2 is not necessarily required for the longevity benefits gained from CR [13]. CR, achieved by growth in 0.2% or less glucose led to a significant increase in RLS, in several *S. cerevisiae* strains and in *C. neoformans* strain, H99 [11,14]. Such CR has also been implicated in affecting PKA and Sch9, which are nutrient-responsive protein kinases shown to regulate RLS in *S. cerevisiae* [15,16]. However, the majority of longevity-mediating genes identified in *S. cerevisiae* are involved in the Target of Rapamycin (TOR) pathway [17].

An earlier study from our laboratory was the first to characterize the contribution of *SIR2* to cryptococcal aging [14]. In that study, *SIR2* was chemically activated or inhibited to modulate the RLS, and subsequently the resilience of *C. neoformans* strain, H99, which is the standard laboratory strain used by most investigators. In this new study, we generated a *sir2Δ* mutant (CNJ02940) in another *C. neoformans* strain, RC2, to test whether this effect was universal for *C. neoformans*. *SIR2* expression, in addition to other genes that influence longevity under CR, was investigated with several low passaged clinical *C. neoformans* strains.

## 2. Materials and Methods 

### 2.1. Ethics Statement

Vertebrate animal experiments were carried out with the approval of the Albert Einstein College of Medicine Institute for Animal Studies (protocol #20091015, approved 29 January 2010), and adhere to any federal, state, local, and institutional guidelines.

### 2.2. Strains

*C. neoformans* strains used in this study are listed in Table S1. Prior to experiments, strains were maintained at –80 °C, then sub-streaked to single colonies at least three times on standard media at 37 °C, including Yeast Extract Peptone (YEP) agar or broth (Difco) with 0.05% or 2% dextrose (Fisher), or isonicotinamide (INAM) as previously described [18]. *Escherichia coli* with plasmids, pJAF1, pJAF13, and pUC19 were grown on Luria Bertani (LB) agar (Fisher, Fair Lawn, NJ, USA) with ampicillin and have been described elsewhere [19].

### 2.3. Growth Curves

*C. neoformans* cells were grown overnight in YEP broth with 2% dextrose at 37 °C with agitation, then diluted to an OD of 0.01, and grown in YEP with 0.05% or 2% dextrose. Optical density was measured every 30 min for 48 h in a Bioscreen-C Automated Growth Curve Analysis System (Growth Curves USA, Piscataway, NJ, USA), and used to generate a growth curve.

### 2.4. Replicative and Chronological Lifespan

RLS was determined by adaptation of a previously published method in *S. cerevisiae* [20]. Briefly, *C. neoformans* cells (*n* = 20–40) for each strain were arrayed for micro-dissection on an agar plate supplemented with YEP and 0.05% or 2% dextrose, or and maintained at 37 °C. The first bud of each cell (virgin mother cell) was followed for each budding event by separating ensuing daughter cells every 1–2 h using a fiber optic needle (Cora Styles, Talent, OR, USA) affixed to the micromanipulator of an Axioscope A1 microscope (Zeiss, Thornwood, NY, USA). Plates were kept at 4 °C overnight to prevent excessive budding, and the experiment was concluded when cells had ceased division for 24 h. RLS was quantitated by summing the buds made by each virgin cell, and reported as the median of all mothers cells examined. Chronological lifespan (CLS), or the length of time non-dividing cells survive, was determined by adaptation of a previously published method in *S. cerevisiae* [21]. Briefly, 10^6^ *C. neoformans* cells/ml were grown in Yeast Peptone Dextrose (YPD) broth at 37 °C with agitation until they reached stationary phase (3 days). All media was then removed by washing in sterile dH_2_O, and cells were maintained in dH_2_O until 99.9% of the starting culture was dead. Viability of the culture was measured every 2 d by viable plate counts of appropriate dilutions on YPD agar.

### 2.5. Disruption and Complementation of the SIR2 Gene

For disruption of the *SIR2* (CNJ02940) gene, primers were designed to its complete open reading frame sequence determined from JEC21 genome to replace it with a neomycin cassette (*NEO*) in wildtype RC2 cells. This was accomplished by homologous recombination using biolistic transformation in a Particle Delivery System-1000/He hepta system (Biorad, Hercules, CA, USA) to deliver a linearized DNA construct containing neomycin under *ACT1* promoter control and a *TRP1* terminator with an additional 1000 bp of up- and downstream regions of the target sequence. *NEO* was amplified from plasmid pJAF1 using primers, Neo-F and Neo-R, and the ampicillin resistance gene was amplified from plasmid pUC19 using primers, pUC19-F and pUC19-R (see Table S2 for all primers used). All primers were designed with a Van91I restriction site for one-step directional cloning, and amplified products were restricted with Van91I and ligated using Quick ligase (New England Biolabs, Ipswich, MA, USA). Products were transformed into XL10 Gold cells (Agilent, Santa Clara, CA, USA), and transformants were selected on LB plates supplemented with ampicillin and confirmed by single digestion with Van91I. Transformants (RC-*sir2Δ*) were linearized using primers, RC2SIR2-Lfor and RC2SIR2-Rrev, and screened on YPD plates supplemented with 100 μg/ml G418 sulfate (neomycin), then further confirmed by PCR (Figure S1). For complementation, Wildtype *SIR2* was amplified with its native promoter from the JEC21 genome using primers, RC2SIR2R-For and RC2SIR2R-Rev, containing Eco RV and Xho I restriction sites. The amplified gene was cloned into plasmid pJAF13, linearized using Apa I, and randomly inserted into *sir2Δ* cells by biolistic transformation. Resulting transformants (RC2-*sir2Δ+SIR2*) were selected on YPD plates containing 100 μg/ml nourseothricin (Werner Bioagents, Jena, Germany), and confirmed by PCR.

### 2.6. Phenotypic Characterization

Cell and capsule sizes were measured on *C. neoformans* cells grown in respective media. Cells were suspended in India ink and imaged (*n* = 100 per group) at 1000× magnification on an Olympus AX70 microscope (Waltham, MA, USA) attached to a Qimaging Retiga 1300 digital camera (Qimaging, Surrey, BC, Canada), and sizes recorded in Adobe Photoshop CS5 (San Jose, CA, USA) for Macintosh. Capsule staining patterns were determined on *C. neoformans* cells stained with mAb 18B7, which is specific to the glucuronoxylomannan component of the capsule, and visualized with fluorescein isothiocyanate-labeled goat anti-mouse immunoglobulin G as previously described [22]. Additional, phenotypic characterization, including switching frequencies, hydrogen peroxide disc diffusion assay, macrophage-mediated phagocytosis and killing, capsule induction, melanization, mating ability, and minimum inhibitory concentrations to various antifungals were determined as previously described by this lab and other groups [14,19,23].

### 2.7. Infection Studies

*Galleria mellonella* infection was performed as previously described [24]. Briefly, a suspension of 2 × 10^4^ *C. neoformans* cells was injected in the proleg of larvae (*n* = 20) (Vanderhorst Wholesale, Inc., St. Mary’s, OH, USA). In addition, 2.5 nM INAM in phosphate buffered saline was injected into a different proleg every 2 days. Murine infection studies were performed as previously described [8]. Specifically, 10^6^ *C. neoformans* cells were injected intravenously or intratracheally into 6–8 week old female BALB/c mice (*n* = 10) (National Cancer Institute, Bethesda, MD, USA).

### 2.8. Transcriptome Analysis

RNA was collected for sequencing as previously published [14]. Differential gene expression and gene ontology enrichment (GO) was performed by the Genome Technology Access Centre at Washington University in St. Louis, MO, USA, and deposited on GEO (accession #GSE74298). Real-time PCR was performed on RNA isolated from strains grown in YEP with 0.05% or 2% dextrose. Briefly, RNA was extracted with the RNeasy mini kit (Qiagen, Hilden, Germany) per manufacturer’s instructions, with an initial step of mechanical disruption using a mini bead beater (Biospec, Bartlesville, OK, USA). RNA was then cleaned for DNA contamination using the MessageClean kit (GenHunter Corp., Nashville, TN, USA), and then real-time PCR was performed using the Power SYBR Green RNA-to-C_T_ 1-Step kit (Applied Biosystems, Foster City, CA, USA) per the manufacturer’s instructions, and using primers listed in Table S3. Expression levels were normalized against β-actin, and transcript levels were determined using the delta–delta CT method by comparing expression at strains grown in 0.05% to 2% dextrose.

### 2.9. Statistics

Statistical analyses, including Student’s *t*-test and Log-rank test, Wilcoxon rank sum test, and Spearman’s rank correlation were performed on Prism version 7 (Graphpad, La Jolla, CA, USA) or Microsoft Excel 2017 for Macintosh. Differences were considered significant if *p* < 0.05. For transcriptome analysis, genes with a *p* < 0.05 by a hypergeometric test, and a False Discovery Rate *q <* 0.25 were termed significant. 

## 3. Results

### 3.1. Calorie Restriction Variably Affects the Replicative Lifespan of C. neoformans

**Figure 1:**
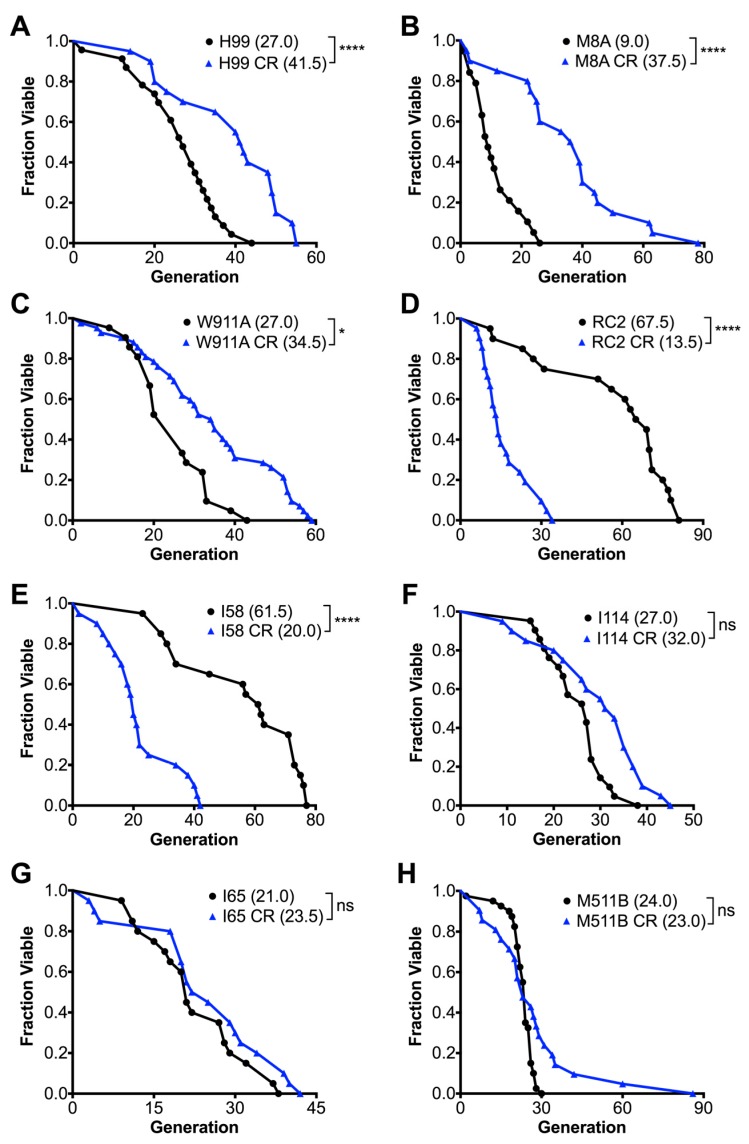
A variable replicative lifespan (RLS) was observed for clinical *C. neoformans* strains grown in calorie restricted (CR) media. Strains grown in CR media were subjected to 0.05% glucose compared to 2% glucose. CR significantly extended the median RLS of (**A**) strain H99 by 54%; (**B**) strain M8A by 317%; and (**C**) strain W911A by 28%. CR significantly shortened the median RLS of (**D**) strain RC2 by 80%, and (**E**) strain I58 by 67%. CR did not significantly affect the median RLS of strains (**F**) I114, (**G**) I65, and (**H**) M511B. Experiments were performed in duplicates with the respective medium (*n* = 20–40 cells). *p*-values were calculated by Wilcoxon Rank Sum test (*p < 0.05, **** *p* < 0.0001, ns not significant).

The effect of calorie restriction (CR) on the RLS of *C. neoformans* was determined in 0.05% glucose growth conditions, which corresponds to the glucose concentration encountered in human cerebrospinal fluid. As expected, CR extended the median RLS of strain H99 by 54% from 27.0 to 41.5 generations (*p* < 0.0001 by Wilcoxon Rank Sum test) (Figure 1A), strain M8A by 317% from 9.0 to 37.5 generations (*p* < 0.0001) (Figure 1B), and strain W911A by 28% from 27.0 to 34.5 generations (*p* < 0.01) (Figure 1C). However, under CR growth conditions, we also found two clinical strains that exhibited a dramatically shortened lifespan; specifically, RC2 cells exhibited a 80% shortened RLS from 67.5 to 13.5 generations (*p* < 0.0001) (Figure 1D), and I58 cells exhibited a 67% shortened RLS from 61.5 to 20.0 generations (*p* < 0.0001) (Figure 1E). In addition, our lifespan analysis demonstrated that some strains had no change in lifespan under CR, including strain I114 (Figure 1F), I65 (Figure 1G), and M511B (Figure 1H). The change in RLS in response to CR was not dependent on whether the strain was *Cryptococcus* var. *neoformans* (serotype D, genotype VNIV) or var *grubii* (serotype A, genotype VNI). Doubling times varied among the strains (Table 1), and were consistently longer or unchanged in CR. However, growth times in CR media were not predictive of RLS (ρ not significant by Spearman rank correlation). In summary, CR extended lifespan in only three out of eight *C. neoformans* strains, and dramatically shortened lifespan in two strains, whereas, in others, it had no effect indicating that *SIR2* expression could be variable and needs to be investigated further.

### 3.2. Loss of SIR2 Has a Significant Effect on the Phenotype of Strain RC2

**Figure 2:**
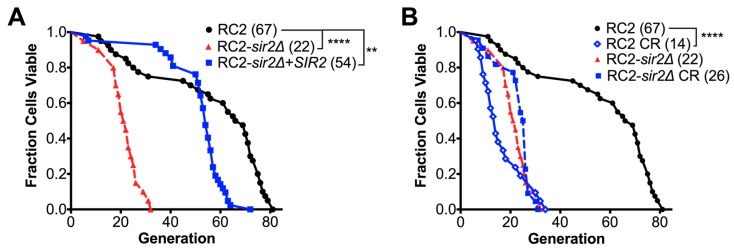
CR mediated loss of replicative lifespan was not mediated or affected by *SIR2* in strain RC2. (**A**) Median RLS of RC2*-sir2Δ* cells was significantly shortened by 68% compared to wt. The loss of lifespan was partially recovered after complementation; (**B**) under CR growth, the loss of lifespan of the *sir2Δ* was comparable to the wt strain. Experiments were performed in duplicates with the respective medium (*n* = 20–40 cells). *p*-values were calculated by Wilcoxon Rank Sum test (** *p* < 0.01, **** *p* < 0.0001).

Given the known role of *SIR2* in replicative aging, and its CR mediated pro-longevity effect in H99 [14,23], and other yeasts [10,11], we investigated its role in CR of strain RC2, which showed such a markedly different response to CR (Figure 1D). First, *SIR2* was deleted by homologous recombination in RC2 (RC2*-sir2Δ*, Figure S1). Additionally, the complemented strain (RC2-*sir2Δ*+SIR2) was generated by non-homologous reconstitution. Loss of *SIR2* in this strain, similar to what we observed in our *sir2Δ* of H99 [14], resulted in mildly attenuated growth in a nutrient rich medium and reduced mating ability (Table 2 and Table S4). Only minor differences in capsule size were noted in the mutant. In addition, only a mildly reduced chronological lifespan (CLS) was noted compared to the wildtype (wt). As expected, median RLS of RC2*-sir2Δ* cells was shortened by 68% from 67.5 to 21.5 generations (*p* < 0.0001 by Wilcoxon Rank Sum test). This was mostly recovered (54.0 generations, *p* < 0.01 by Wilcoxon Rank Sum test) after complementation (Figure 2A). In addition, loss of lifespan (about 67%) of the *sir2Δ* was comparable to that of the RC2 wt strain under CR growth conditions (Figure 2B), indicating that the CR mediated loss of lifespan in wt RC2 was not mediated or affected by *SIR2* function.

**Figure 3:**
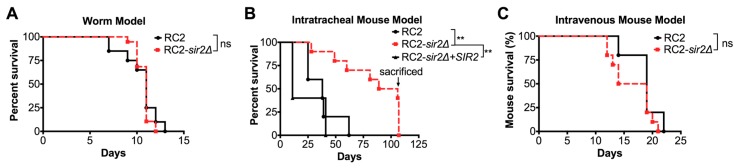
*SIR2* loss affected virulence in some infection models. (**A**) *Galleria mellonella* larvae infected with 2 × 10^4^ RC2-*sir2Δ* cells demonstrated no significant survival difference compared to those infected with wt cells (ns by Log Rank test); (**B**) BALB/c mice infected intratracheally with 10^6^ RC2-*sir2Δ* cells survived significantly longer than those infected with the wt and complemented strains (97.5 versus 38 and 11 days median, *p* < 0.01 by Log Rank test); (**C**) BALB/c mice infected intravenously with 10^6^ RC2-*sir2Δ* cells demonstrated no significant survival difference compared to those infected with wt cells (median survival 16.5 versus 19 days in mutant vs. wt, ns by Log Rank test). Experiments were performed in duplicates (*n* = 10 mice). *p*-values were calculated by Wilcoxon Rank Sum test (** *p* < 0.01, ns not significant).

As expected from our studies with H99, loss of *SIR2* affected some virulence. Waxmoth larvae (*Galleria mellonella*) infected with 2 × 10^4^ RC2-*sir2Δ* cells demonstrated no survival difference compared to those infected with wt cells (Figure 3A). RC2-*sir2Δ* cells were less virulent compared to the respective wt and complemented strains (97.5 versus 38 and 11 days median survival, *p* < 0.01 by Log Rank test) (Figure 3B) in BALB/c mice infected intratracheally with 10^6^ cells. A trend, albeit not significant, of lower virulence was also observed when BALB/c mice that were infected intravenously (median survival 16.5 versus 19 days in RC2-*sir2Δ* vs RC2-wt, respectively, ns by Log Rank test, Figure 3C). Furthermore, doubling times of wt and mutant were comparable in vitro in the low glucose growth conditions (Table 2) mimicking the brain environment. These data further supported the notion that CR did not have a life prolonging effect on RC2 and was not mediated by *SIR2*.

### 3.3. Gene Regulation of C. neoformans under Calorie Restriction

**Figure 4:**
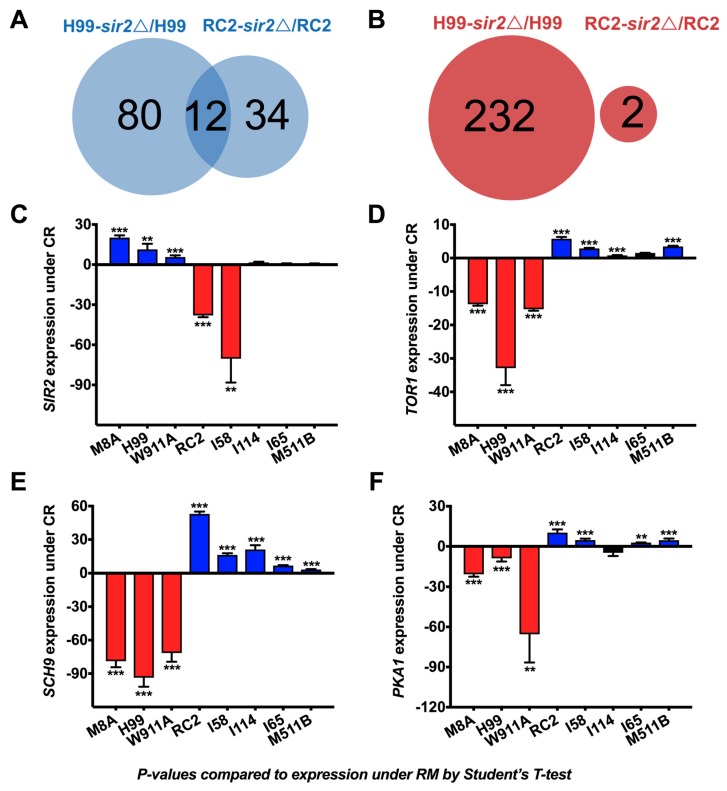
Genes were differentially regulated in *C. neoformans* strains grown under calorie restriction. Transcriptome analysis of genes regulated under *SIR2* for strains grown in CR revealed that (**A**) 10.5% (12 out of 114) upregulated genes were common to strains H99 and RC2, and (**B**) 0% (0 out of 234) downregulated genes were common to strains H99 and RC2. RT-PCR analysis of several *C. neoformans* strains grown in CR revealed that (**C**) *SIR2* expression was significantly up in strains H99, M8A, and W911A cells, and significantly down in strains RC2, and I58. No significant regulation was found in strains I114, I65, and M511B. (**D**) *TOR1*, (**E**) *SCH9,* and (**F**) *PKA1* expression were consistently and significantly down in strains H99, M8A, and W911A cells, and consistently and significantly up in strains RC2, I58, and M511B. Some differences were found in strains I114 and I65, where expression was either significantly up or not significantly affected. For RT-PCR analysis, experiments were performed in quadruplicates and compared to strains grown in rich media. *p*-values were calculated by Student’s *t*-test (** *p* < 0.01, *** *p* < 0.001, ns not significant).

The striking difference of CR on the RLS of cryptococcal strains, H99 and RC2, led us to compare their transcriptional response when grown in CR conditions using the available dataset published by our group (GEO accession #GSE74298). Under CR, the two strains showed markedly different transcriptional responses. We previously reported in the *sir2Δ* of the H99 strain that 340 genes/presumed genes were significantly regulated: 80 were upregulated, and 232 were downregulated under CR. In contrast, a significantly lower number of genes were regulated in the RC2-*sir2Δ*. Here, we found that 34 were upregulated (Figure 4A), and two were downregulated (Figure 4B) under CR. Interestingly, there was very little overlap between the two mutants consistent with their divergent response to CR (Table S5). Gene ontology analysis revealed that, in RC2, the most enriched genes were involved in translation in both rich and CR media regardless of the activity of *SIR2*; however, under CR and loss of *SIR2*, transcription and DNA replication was the most disrupted.

For specific genes that are associated with aging, the transcriptome analysis was confirmed by real-time PCR. Specifically, we showed that *SIR2* was highly (11.4-fold) upregulated in H99 cells (*p* < 0.01), whereas it was 38-fold downregulated (*p* < 0.001) in RC2 cells (Figure 4C). *SIR2* expression was also significantly up in strains M8A (20.2-fold) and W911A (5.7-fold), where CR had a pro-longevity effect on lifespan, and was significantly down in strain I58 (70.4-fold), where CR had a detrimental effect on lifespan. No significant regulation was observed in strains I114, I65, and M511B, where CR had no significant effect on lifespan. Similarly, the activity of *TOR1, PKA1, SCH9* was differential among the examined strains. *TOR1* expression was significantly down in strains M8A, H99, and W911A, and significantly up in strains RC2, I58, I114, and M511B (Figure 4D). *SCH9* expression was significantly down in strains M8A, H99, and W911A, and significantly up in the remaining strains (Figure 4E). *PKA1* expression was significantly down in strains M8A, H99, and W911A, and significantly up in strains RC2, I58, I65, and M511B (Figure 4F). In summary, comparative transcriptional analysis confirms the differential regulation of *SIR2* among other genes in response to CR in *C. neoformans* strains.

### 3.4. SIR2 Is Differentially Regulated in RC2

**Figure 5:**
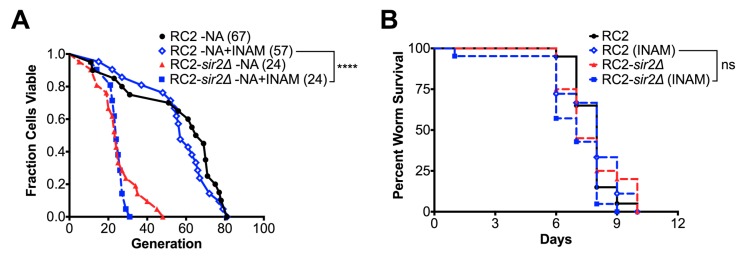
The *SIR2* modifying drug, isonicotinamide, did not affect the lifespan or virulence of strain RC2. (**A**) RLS of the *sir2*Δ and wt cells of RC2 was not significantly different when grown in media supplemented with 25mM INAM; (**B**) repeated 2.5 mM INAM treatment did not affect the survival of *Galleria mellonella* infected with *sir2*Δ or wt cells. Experiments were performed in duplicates (*n* = 20–40 cells or larvae). *p*-values were calculated by Wilcoxon Rank Sum test or Log Rank test (**** *p* < 0.0001, ns not significant).

Isonicotinamide (INAM) and other *SIR2* modifying drugs have been shown to extend RLS in *S. cerevisiae* [18], and most importantly in *C. neoformans* strain, H99 [14], through the action of *SIR2*. *C. neoformans* grows in the human host in the brain niche, where lower glucose concentrations are present. Hence, these drugs do not change RLS when *SIR2* is mutated. Given the finding that *SIR2* is differentially regulated, we tested INAM’s effect on the RLS and resilience of strain RC2, where CR failed to extend RLS (Figure 1D), and the transcriptome was markedly different in the *sir2Δ* and wt in response to CR (GEO accession #GSE74298). In vitro, 25 mM INAM had no effect of on the lifespan of *sir2Δ* or wt cells (Figure 5A). We also investigated if the drug could alone affect resilience in the *Galleria* model. Repeated 2.5 mM INAM treatment alone had no effect; larvae injected only with INAM had comparable survival to those treated with PBS as established previously [14]. As expected, INAM treatment in vivo also had no effect on survival of RC2-infected larvae (Figure 5B). Taken together, our data indicate that *SIR2* regulation in *C. neoformans* is not consistent in all strains, and therefore *SIR2* modulating drugs may also not consistently have an effect on the outcome of infection.

## 4. Discussion

This study investigates the role of *SIR2* in the context of calorie restriction and its implications for the replicative lifespan of *C. neoformans*. Glucose utilization to repress aerobic respiration is a characteristic of Crabtree positive yeasts, such as *S. cerevisiae* and *S. pombe* [25]. Unlike them, *C. neoformans* and other pathogenic yeasts, including *Candida species* are Crabtree negative, and prefer respiration to fermentation regardless of glucose availability [26]. Low glucose growth conditions are present in the spinal fluid from which *C. neoformans* strains are commonly recovered. Therefore, the basidiomycete, *C. neoformans* serves as a unique model for studies in aging and CR, which are primarily done in ascomycetes. Current findings in *C. neoformans* are limited: a study by our laboratory [14] has shown that *SIR2* modulating drugs change the replicative lifespan of a *C. neoformans* population, and by shifting its median generational age, and change its vulnerability to antifungal drugs, which are more effective on young yeast cells. Early studies in *S. cerevisiae* show that glucose restriction to 0.05% increases RLS by 20–40% [13,15,27], and this was attributed to *SIR2* [28]. However, upon further exploration, the link between *SIR2* and CR has been called into question, even in *S. cerevisiae* [13]. Therefore, we took a cautious approach in investigating this in *C. neoformans*, where *SIR2* remains highly conserved [11,14].

As shown in the widely studied *S. cerevisiae*, several genes are also affected by CR in *C. neoformans*. The most prominent signature associated with aging is *SIR2*, which showed differential regulation in two well-studied *C. neoformans* strains, H99 and RC2. Both strains are highly passaged, which has been shown to result in the emergence of genotypic and phenotypic variants [29,30]. While more is known about the function of *SIR2* in H99 based on a study from our group [14], not much is known in RC2, or low passaged clinical strains. RC2 was of particular interest because it undergoes phenotypic switching to a hypervirulent mucoid phenotype [30,31,32], which has a shorter RLS [8], confirming that lifespan can be regulated. The relationship between phenotypic switching and loss of RLS has also been found in *Candida glabrata* and is associated with poor outcome for both fungal infections [9,33].

Variation in Sir2 activity stemming from strain differences has been described in *S. cerevisiae* [13], and we confirmed this in *C. neoformans*. Two laboratories have investigated different aspects of *SIR2* function in H99 [14,23]. Our study investigated the effect of *SIR2* modulating drugs on H99, whereas Arras et al. investigated five classes of sirtuins—*SIR2*, *HST2*, *HST3*, *HST4*, *HST5*—through systematic deletion in H99. Interestingly, they could not reconstitute the loss of the *SIR2* function, and proposed that temporary inactivation of a sirtuin is a mechanism for *C. neoformans* to rapidly undergo heritable microevolutionary changes without altering its genetic material. Although both studies focused on different aspects, we found similarities and differences in several phenotypes between our H99 *sir2Δ*, and the mutant of the other study.

Our data now support the concept that *SIR2* function varies in strains. Specifically, we found that *SIR2* is important for mating, lifespan, and growth in strain RC2, which has been described in other yeasts [10,13,18], and is consistent with its role in strain H99 as confirmed by our group [14]. Notably, in the study by Arras et al. [23], *C. neoformans* virulence associated traits were only affected by two of the five sirtuins. This is similar to our findings, where the mutant did not have a drastically altered capsule size under non-inducing conditions, and a similar response as the wt to melanization, and minimum inhibitory concentration to amphotericin B. Our data add to their findings by showing that the mutant resists stress as well as the wt, including hydrogen peroxide stress, macrophage-mediated phagocytosis and killing, and growth at 39 °C in a different genotype. Interestingly, no mating defect or growth difference was reported in the Arras study for H99 *sir2Δ*, whereas we found that both the RC2 and the H99 [14] mutant grow slower in YPD; this difference may stem from the choice of method (OD measurements over 48 hours compared to overnight spot dilution), or the H99 variant. Both studies implicated *SIR2* in virulence in different pulmonary murine infection models. In invertebrate models, *Caenorhabditis elegans* and *Galleria mellonella*, no survival difference was observed*.* The function of *SIR2*, and potentially compensatory sirtuins will need to be further investigated in low passaged strains, although previous data indicate that they do not regulate each other [23]. It will be of interest to investigate their function in strain RC2, where variants from microevolutionary pressures in the host emerge and alter the outcome of infection [30,31,32].

In our study, *SIR2* expression was upregulated in strains that had a pro-longevity response to CR (H99, M8A, W911A), indicating its importance to longevity in *C. neoformans*. This was not the case for strains where CR led to loss of lifespan (RC2, I58); here, *SIR2* expression was significantly down, whereas *SIR2* expression was unchanged when CR had no effect on lifespan (I114, I65, M511B). For *C. neoformans*, *SIR2* is of importance because of its role in silencing and because inherited epigenetic modifications, such as histone deacetylation, may affect the outcome of cryptococcal infection [10]. It is difficult to consolidate these findings with Arras et al. [23] since they performed a proteomic analysis on the mutants. Nonetheless, our GO analysis reveals that *SIR2* has a categorical effect on carbohydrate metabolism for H99. This is now confirmed for RC2, importantly in CR growth conditions, thus providing additional insight to the role of *SIR2* in this Crabtree negative yeast.

In addition, of interest was the differential transcription of *TOR1*, *PKA*, and *SCH9*, which in *S. cerevisiae* are regulated under CR and also associated with changes in RLS [28,34,35,36]. Deletion of *TOR1* in *S. cerevisiae* [37,38] results in 20% increased mean RLS; in *C. neoformans*, *TOR1* has been suggested to be essential [39]*.* *PKA* and *SCH9* have been shown to regulate RLS in *S. cerevisiase* [15,16]; specifically, a decrease in PKA activity results in an increased RLS; TOR in comparison acts upstream and parallel to PKA, whereas Sch9 acts parallel to both PKA and TOR [34,36]. Epistatic experiments involving *FOB1* suggest that decreased activity of Sch9 and TOR in response to CR results in increased RLS in *S. cerevisiae* [13]. These studies also showed that, similar to CR, decreased TOR activity is a strain-independent mechanism to increase longevity in *S. cerevisiae*.

Accordingly, all three genes were significantly downregulated, in strains where CR prolonged lifespan (M8A, H99, W911A). In strains where CR had a detrimental effect (RC2, I58), all were significantly upregulated. In strains where CR had no considerable influence on lifespan (I114, I65, M511B), these genes were for the most part significantly upregulated, or not significantly regulated. This upregulation likely stems from their involvement in important regulatory processes relating to metabolism, translation, and ribosome biogenesis. In *S. cerevisiae*, all consistently regulate the expression of ribosomal proteins [35,40], which may also be true for *C. neoformans*. In summary, comparative transcriptional analysis confirms the differential regulation of several genes implicated in CR in *S. cerevisiae* with *C. neoformans*. It will be of interest to study specific genes downstream of *TOR* that have been implicated in CR, namely *Gln3* and *Rom2* [37,38,41]. Ure2, regulates the nitrogen-responsive transcription factor Gln3, and an activator of protein kinase C, Rom2. Their homologs are implicated in *C. neoformans* virulence, and deletion mutants exist [42,43]. It will also be of interest to study the genes commonly regulated under CR in the mutants of both RC2 and H99 background. Not surprisingly, since *SIR2* is NAD-dependent, those involved in nicotinamide metabolism were in common: CNA07130 (dehydrogenase NAD(P)^+^), CNI02360 (NADPH dehydrogenase), and CNA05810 (NAM permease). Other genes in common are implicated in *C. neoformans* biology; CNK02730 (sugar transporter) is responsible for intron loss and *C. neoformans* lineage [44]. Some genes are directly implicated in pathogenesis: CNF04430 (antiphagocytic protein) [45]; CNE02570 (succinate-fumarate antiporter), CNH02190 (malate synthase), and CNI01560 (sterol-binding protein) in early pulmonary infection [46]; the latter hints at the marked attenuated virulence in the pulmonary infection model [14,23]. Other genes include CNJ02210 (spermine transporter) in deubiquitinating [47], and oxygen sensing and sterol homeostasis [48], and CNM00600 (galactose metabolism related protein) discovered under the regulation of *NRG1* in cAMP signaling [49] is implicated in capsule formation and mating.

Our investigation into chemical modulation with isonicotinamide to stimulate *SIR2* revealed differences compared to H99 [14], which highlight the variability of *SIR2* function. While this pleiotropic phenomenon is disappointing from a therapeutic standpoint, it may not be entirely surprising as such variability of RLS in response to nutrients has been described in *S. cerevisiae* [11], where the direct role of *SIR2* in response to CR has been strain-dependent. Nonetheless, our findings and the available literature on *SIR2* in *C. neoformans* [14,23] suggest that *SIR2* activity may have evolved differently in this yeast, which, opposed to *Saccharomyces*, has to adapt to very different host environments [26]. Further research is needed to explore the relationship of stress, generational age, and *SIR2* transcription in *C. neoformans* strains, and how these factors alter their epigenetic landscape. It seems prudent to validate any conclusions in low passaged clinical strains, even environmental strains where virulence is attenuated, and future work will need to clarify the role of genes that may compensate for *SIR2* function in both highly passaged and low passaged strains.

## Figures and Tables

**Table 1:** Doubling times of *C. neoformans* strains in rich media (RM) and calorie-restricted media (CR).

Strain	Doubling Time in RM (h)	Doubling Time in CR (h)	*P*-Value
H99	2.1 ± 0.3	2.8 ± 0.2	0.01
M511B	3.1 ± 0.2	3.6 ± 0.3	0.01
M8A	2.4 ± 0.3	2.2 ± 0.2	ns
I58	2.5 ± 0.3	2.9 ± 0.4	ns
I65	2.6 ± 0.2	3.0 ± 0.3	0.05
I114	2.3 ± 0.1	4.0 ± 0.2	0.01
RC2	2.4 ± 0.2	3.0 ± 0.2	0.01
W911A	3.4 ± 0.3	3.6 ± 0.3	ns

**Table 2:** Characterization of *sir2Δ* phenotypes in *C. neoformans* strain RC2.

Phenotype	WT	Mutant	*p*-Value
Doubling time in YPD	2.0 ± 0.2 h	2.8 ± 0.3 h	< 0.01
Doubling time in 0.05% YPD	3.0 ± 0.2 h	3.4 ± 0.4 h	ns
Mating in V8 agar	No with Kn99*MATa*	No with Kn99*MATa*	ns
Chronological lifespan	19 d	21 d	ns
Phenotypic switching rate	0.5 × 10^−4^	0.5 × 10^−4^	ns
Uninduced capsule size	1.48 ± 0.34 µm	2.19 ± 0.28 µm	< 0.01
Induced capsule size	5.89 ± 1.29 µm	5.65 ± 0.92 µm	ns
Total cell size	7.25 ± 1.09 µm	9.16 ± 0.83 µm	< 0.01
Phagocytosis index	20.32%	31.67%	ns
Killing in macrophages	59.06%	69.36%	ns
Colony sectoring	No sectoring	No sectoring	ns
Melanization	Same degree	Same degree	ns
GXM stain (18B7 mAb)	Same pattern	Same pattern	ns
MIC to amphotericin B	0.125 µg/mL	0.125 µg/mL	ns
H_2_O_2_ resistance	3.1 cm	3.0 cm	ns
39 °C growth	Grows	Grows	ns

GXM: Glucuronoxylomannan, MIC: Minimum Inhibitory Concentration

## Supplementary Materials

The following are available online at http://www.mdpi.com/2309-608X/4/1/26/s1. Figure S1: Confirmation of the *sir2Δ* mutant; Table S1: Strains used in the study; Table S2: List of primers used in the construction of the *sir2Δ* mutant; Table S3: List of primers used to measure mRNA expression of select genes; Table S4: Comparison of the *sir2Δ* phenotypes in the H99 and RC2 strains; Table S5: List of differentially regulated genes in RC2-*sir2Δ* compared to RC2 cells grown in calorie restricted media.
