# Supplementary material for: Differences in Sirtuin Regulation in Response to Calorie Restriction in Cryptococcus neoformans
Source: J Fungi (Basel). 2018 Feb 18;4(1):26. doi: 10.3390/jof4010026 (PMC5872329; doi:10.3390/jof4010026)
Supplement: Supplementary file 1 [file jof-04-00026-s001.docx]

Differences in Sirtuin Regulation in Response to Calorie Restriction in *Cryptococcus neoformans*

Tejas Bouklas ^1,^*, Lindsey Masone ^1^ and Bettina C. Fries ^2^

^1^ Department of Biomedical Sciences, Long Island University-Post, Brookville, NY 11548, USA; lindsey.masone@my.liu.edu

^2^ Department of Medicine (Division of Infectious Diseases) and Department of Molecular Genetics and Microbiology, Stony Brook University, Stony Brook, NY 11794, USA; bettina.fries@stonybrookmedicine.edu

***** Correspondence: tejas.bouklas@liu.edu; Tel.: +91-516-299-2695

**Table S1.** Strains used in the study.

| ***C. neoformans* strains** | **Serotype** | | **Source or Reference** |
| --- | --- | --- | --- |
| H99 | A | J. Perfect, Duke University, USA | |
| H99-*sir2Δ* | A | [1] | |
| RC2 | D | ATCC 24067 variant | |
| RC2-*sir2Δ* | D | This study | |
| RC2-*sir2Δ+SIR2* | D | This study | |
| I58 | A | [2], India | |
| I65 | A | [2], India | |
| I114 | D | [2], India | |
| M8A | A | [3], Montefiore Medical Center, USA | |
| M511B | A | [3], Montefiore Medical Center, USA | |
| W911A | A | [3], Montefiore Medical Center, USA | |

**Table S2.** List of primers used in the construction of the *sir2Δ* mutant.

| **Name** | **Purpose** | **Sequence (5’–3’)** |
| --- | --- | --- |
| Neo-F  Neo-R | Amplification of neomycin cassette | CCATATGTTGGTAA AACGACGGCCAGTGAATTGTA  CCATGAATTGGCAGGAAACAGCTATGACCATGATT |
| pUC19-F  pUC19-R | Amplification of origin of replication and ampicillin resistance gene | CCATTTTTTGGGAAAGGGCCTCGTGATACGCCT  CCATTCTTTGGGCTTTCCAGTCGGGAAACCTGT |
| RC2SIR2-Lfor  RC2SIR2-Lrev | Amplification of 1000 nucleotides upstream of *SIR2* in RC2-wt | CCATAGATTGGCCGTTCTCCTTTGTTGATCG  CCATCATTTGGGGAATAATGGATGAAGCGAGA |
| RC2SIR2-Rfor  RC2SIR2-Rrev | Amplification of 1000 nucleotides downstream of *SIR2* in RC2-wt | CCATTTCTTGGTTTTTCTTTTCCGCCTAATCTTC  CCATAAATTGGAGATGTATGTATGATGGGGTGGT |
| RC2SIR2R-For  RC2SIR2R-Rev | Complementation of *SIR2* in RC2-wt | GATATCGCCGCTTCCCAAACATAAT  CTCGAGTGCAGACATGGCAGGAATAG |

**Table S3.** List of primers used to measure mRNA expression of select genes.

| **Primer name** | **Gene name** | **Sequence (5’–3’)** |
| --- | --- | --- |
| CNJ02940For  CNJ02940Rev | *SIR2* (CNJ02940) | CCAAGTCTAAGAAGATCATT  CCAAGTCTAAGAAGATCATT |
| CNF03740For | *TOR1* (CNF03740) | AGCATCGCAAGACACTGGAA |
| CNF03740Rev |  | ATTGGAAAGGAGCTGACGGG |
| CNN00360For | *SCH9* (CNN00360) | GCGTAGTCAGCACCAGAACT |
| CNN00360Rev |  | CCAGAGTCTCTCAAAGCGCA |
| AF481770For  AF481770Rev | *PKA1* (AF481770) | GACGTCCGTCCCTGTTAGTG  ACTTATACGGTCGTCACCGC |

**Table S4.** Comparison of the *sir2Δ* phenotypes in the H99 and RC2 strains.

| **Phenotype** | **Phenotype in mutant compared to WT for RC2 ^1^** | **Phenotype in mutant compared to WT for H99 ^2^** |
| --- | --- | --- |
| **Doubling time in YPD** | Longer | Longer |
| **Doubling time in 0.05% YPD** | No measured difference | No measured difference |
| **Mating in V8 agar** | No with Kn99*MATa* | No with Kn99*MATa* |
| **Chronological lifespan** | No measured difference | Decreased |
| **Phenotypic switching rate** | Same as WT | No switching in mutant or WT |
| **Uninduced capsule size** | Decreased | Decreased |
| **Total cell size** | Smaller | No measured difference |
| **Phagocytosis index** | No measured difference | No measured difference |
| **Killing in macrophages** | No measured difference | No measured difference |
| **Colony sectoring** | No sectoring in mutant or WT | No sectoring in mutant or WT |
| **GXM stain (18B7 mAb)** | Same pattern | Same pattern |
| **MIC to amphotericin B** | No measured difference | No measured difference |
| **H_2_O_2_ resistance** | No measured difference | No measured difference |

^1^ Supporting information in Table 2, ^2^ Supporting information in publication [[1](#_ENREF_1)].

**Table S5.** List of differentially regulated genes in RC2-*sir2Δ* compared to RC2 cells grown in calorie restricted media. Genes in common with transcriptome analysis in H99 are marked with an asterisk. Genes with a fold change > 1.5, *p* < 0.05, and FDR *q* < 0.25 are considered significant.

| **Gene ID** | **Function** | **Fold change (*Δ*/WT)** |
| --- | --- | --- |
| CNH03030 | hypothetical protein | 3.7756 |
| CNI01750 | expressed protein | 2.94209 |
| CNE00960 | conserved hypothetical protein | 2.78407 |
| CNK02730* | sugar transporter, putative | 2.76272 |
| CNA07130* | succinate-semialdehyde dehydrogenase [NAD(P)+], putative | 2.70732 |
| CNL06180 | class III aminotransferase, putative | 2.56992 |
| CNE02620 | branched-chain alpha-keto acid dehydrogenase E1-alpha subunit, putative | 2.42506 |
| CNE00400* | expressed protein | 2.42248 |
| CNB01020 | mandelate racemase/muconate lactonizing enzyme, putative | 2.25019 |
| CNI02040 | dihydrodipicolinate synthase, putative | 2.2392 |
| CNC04510 | conserved hypothetical protein | 2.21415 |
| CNA05810* | nicotinamide mononucleotide permease, putative | 2.15096 |
| CNF04430* | antiphagocytic protein, putative | 1.96956 |
| CNN00270 | deacetylase, putative | 1.92535 |
| CNJ03160 | conserved hypothetical protein | 1.90551 |
| CNN00670 | expressed protein | 1.8514 |
| CNA07050 | hypothetical protein | 1.84546 |
| CNE02570* | succinate:fumarate antiporter, putative | 1.82598 |
| CNM00600* | galactose metabolism-related protein, putative | 1.81386 |
| CNA05220 | membrane transport protein, putative | 1.81172 |
| CNL04570 | conserved hypothetical protein | 1.78757 |
| CNC06420 | conserved hypothetical protein | 1.7853 |
| CNJ02210* | spermine transporter, putative | 1.75303 |
| CNI01300 | 2,4-dichlorophenoxyacetate alpha-ketoglutarate dioxygenase, putative | 1.73128 |
| CNH02910* | malate synthase, putative | 1.72961 |
| CNC03730 | conserved hypothetical protein | 1.72439 |
| CNA04160 | transporter, putative | 1.65417 |
| CNI02360* | NADPH dehydrogenase 2, putative | 1.61274 |
| CNN00760* | hypothetical protein | 1.60334 |
| CNJ01570 | hypothetical protein | 1.59395 |
| CNI00360 | oxidoreductase, putative | 1.58147 |
| CNF04760 | conserved hypothetical protein | 1.54809 |
| CNI01560* | sterol-binding protein | 1.53559 |
| CNA07920 | siderochrome-iron (ferrioxamine) uptake transporter, putative | 1.53388 |
| CNE02900 | conserved hypothetical protein | -2.07138 |
| EFCNEG00000000049 | tRNA-Glu for anticodon CUC [Source:TRNASCAN_SE;Acc:tRNA-Glu] | -2.22803 |


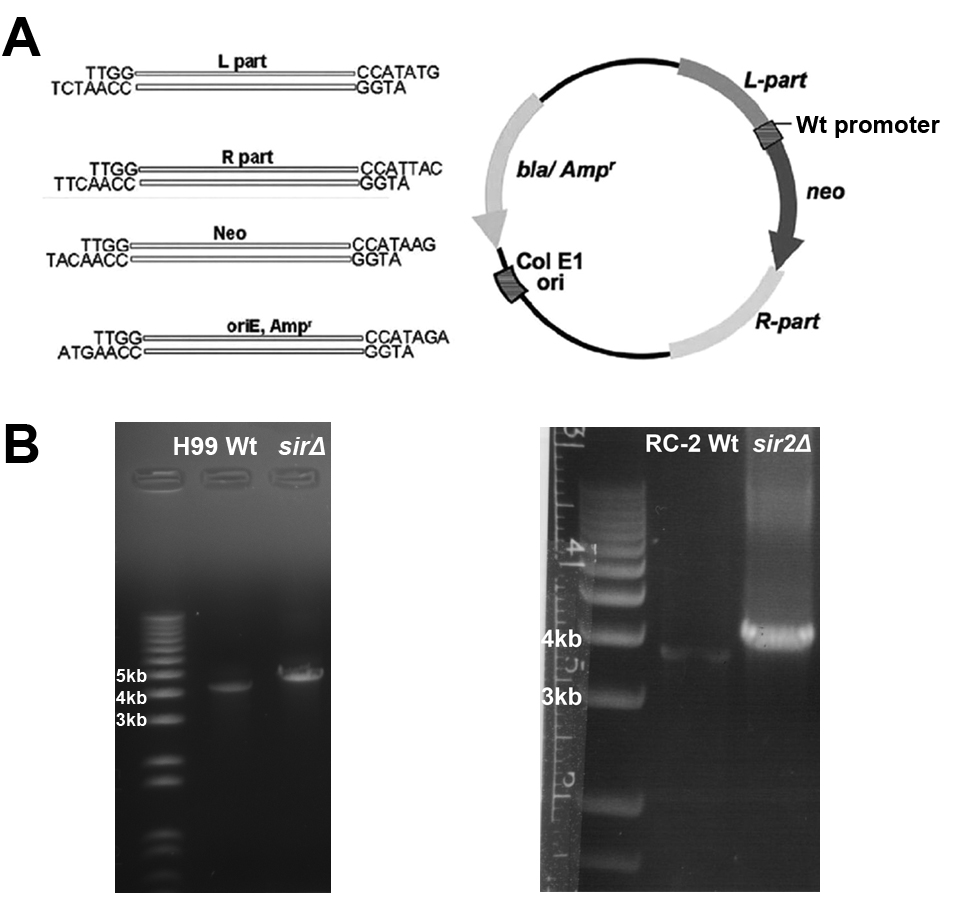


**Figure S1.** Confirmation of the *sir2Δ* mutant. PCR amplification showing correct homologous recombination in the mutant (*sir2Δ*, second lane) compared to the wt strain, RC2, (first lane).

References

1. Bouklas, T.; Jain, N.; Fries, B.C. Modulation of replicative lifespan in cryptococcus neoformans: Implications for virulence. *Front Microbiol* **2017**, *8*, 98.

2. Jain, N.; Wickes, B.L.; Keller, S.M.; Fu, J.; Casadevall, A.; Jain, P.; Ragan, M.A.; Banerjee, U.; Fries, B.C. Molecular epidemiology of clinical cryptococcus neoformans strains from india. *Journal of clinical microbiology* **2005**, *43*, 5733-5742.

3. Bouklas, T.; Pechuan, X.; Goldman, D.L.; Edelman, B.; Bergman, A.; Fries, B.C. Old cryptococcus neoformans cells contribute to virulence in chronic cryptococcosis. *MBio* **2013**, *4*.

© 2018 by the authors. Licensee MDPI, Basel, Switzerland. This article is an open access article distributed under the terms and conditions of the Creative Commons Attribution (CC BY) license (http://creativecommons.org/licenses/by/4.0/).
